# Ventilator-Associated Tracheobronchitis: To Treat or Not to Treat?

**DOI:** 10.3390/antibiotics9020051

**Published:** 2020-01-31

**Authors:** Despoina Koulenti, Kostoula Arvaniti, Mathew Judd, Natasha Lalos, Iona Tjoeng, Elena Xu, Apostolos Armaganidis, Jeffrey Lipman

**Affiliations:** 1 UQ Centre for Clinical Research, Faculty of Medicine, The University of Queensland, Brisbane 4029, Australia; Mathew.judd@health.qld.gov.au (M.J.); Nlalos@wustl.edu (N.L.); iona.tjoeng@gmail.com (I.T.); elena.xu@uq.net.au (E.X.); j.lipman@uq.edu.au (J.L.); 2 2nd Critical Care Department, ‘Attikon’ University Hospital, Athens 11632, Greece; aarmag@med.uoa.gr; 3 Department of Critical Care Medicine, ‘Papageorgiou’ General Hospital of Thessaloniki, Thessaloniki 56429, Greece; arvanitik@hotmail.com; 4 Department of Intensive Care Medicine, Royal Brisbane and Women’s Hospital, Brisbane 4029, Australia; 5 Royal Brisbane Clinical Unit, Faculty of Medicine, The University of Queensland, Brisbane 4029, Australia

**Keywords:** ventilator-associated tracheobronchitis, VAT, diagnosis, treatment, inhaled antibiotics

## Abstract

Ventilator-associated tracheobronchitis (VAT) is an infection commonly affecting mechanically ventilated intubated patients. Several studies suggest that VAT is associated with increased duration of mechanical ventilation (MV) and length of intensive care unit (ICU) stay, and a presumptive increase in healthcare costs. Uncertainties remain, however, regarding the cost/benefit balance of VAT treatment. The aim of this narrative review is to discuss the two fundamental and inter-related dilemmas regarding VAT, i.e., (i) how to diagnose VAT? and (ii) should we treat VAT? If yes, should we treat all cases or only selected ones? How should we treat in terms of antibiotic choice, route, treatment duration?

## Highlights

- Diagnosis of ventilator-associated tracheobronchitis (VAT) remains unclear. Better diagnostic criteria necessary to avoid under/over-diagnosis.
- Novel molecular biomarkers and lung ultrasound (LUS) may help increase diagnostic sensitivity of VAT. However, further research into these methods is needed.
- To treat or not to treat: a continuing debate surrounding the uncertainties of the cost/benefit of antibiotic treatment of VAT.
- Controversial use of nebulized/aerosolized antibiotics for VAT in the face of limited evidence of their efficacy.
- Could treating VAT reduce ventilator-associated pneumonia (VAP) and its increased risk of mortality, mechanical ventilation (MV) duration and intensive care unit length of stay (ICU-LOS)? A reasonable assumption that still needs further clarification.

## 1. Introduction

Ventilator-associated tracheobronchitis (VAT) refers to a lower respiratory infection of intubated mechanically ventilated patients with no radiological infiltrate present [1]. It has been described in studies on nosocomial respiratory tract infections in the intensive care unit (ICU) since the 1990s [2], however, it was not until the early 2000s that our knowledge of VAT broadened to include increased recognition of its incidence, causative organisms, and impact on patient outcomes [3,4]. Although the reported incidence of VAT varies, most studies has shown that is a frequent complication affecting more than 10% of invasively ventilated ICU patients [3,5,6,7,8,9,10,11,12,13,14,15,16].

Despite the progress, diagnosis of VAT remains challenging and, consequently, treatment decisions remain a challenge as well. While most studies have found VAT itself is not associated with increased mortality [3,5,6,8], several studies suggest that VAT is associated with increased duration of mechanical ventilation and ICU length of stay (LOS) [3,6,8,9,10,11,14], with presumptive consequences increased healthcare costs. Moreover, it has been reported that appropriate systemic antibiotic treatment was associated with reduced progression to ventilator-associated pneumonia (VAP) [5,9,11,17,18]. On the other hand, controversial findings about the duration of mechanical ventilation, length of ICU stay or mortality with antibiotic treatment versus placebo, complicate clinical decision making of VAT treatment in routine clinical practice [5,19].

The lack of a clear and widely accepted definition for diagnosis of VAT limits the potential for clinical research and hampers efforts of researchers and clinicians to adequately recommend guidelines for the diagnosis and treatment of VAT. The aim of this review is to discuss the fundamental and inter-related dilemmas—conflicting research and recommendations—surrounding VAT diagnosis and treatment. We will discuss and critique: (i) Diagnostic approaches and possible under- and over-diagnosis of VAT, and (ii) when to treat (and how) versus when not to treat.

## 2. Diagnosis

While our understanding of VAT pathophysiology has deepened significantly over the last two decades, several questions remain unanswered. All the pathogenetic theories agree that colonization of the lower respiratory tract (LRT) is the initial event of ventilator-associated lower respiratory tract infections (VARI). During ICU admission the oropharyngeal cavity primarily and the stomach secondarily, becomes colonized by endogenous flora and exogenous bacteria acquired by the ICU environment (such as hands/garments of healthcare workers, equipment, water) [20]. Disabled upper airway reflexes due to the endotracheal tube (ETT), constant accumulation of oropharyngeal and/or gastric secretions above the ETT cuff, subsequent microaspiration through microscopic folds of the cuff and downwards migration of those secretions around the external surface of the tube, are well recognized contributing factors of VARI development [20]. Biofilm formation within the ETT and further relocation to the distal airways via the mechanical ventilation cycles has been considered also, as a possible contributing pathogenetic pathway for VARI. The biofilm impedes antibiotics to act and increases colonization and VARI development likelihood [21]. Depending on the type, virulence and inoculum of bacteria as well as their interaction with the host defense, colonization, VAT or VAP develops over time [22]. The main pathogenetic theories can be summarized as follows: a) colonization leads to VAT and VAT leads to VAP, i.e., VAP is preceded by VAT, b) colonization may lead to either VAT or VAP, without VAT being a precursor of VAP, and c) colonization leads to ventilator-associated respiratory infections with some overlap between VAT and early-VAP [4].

Two main diagnostic approaches of VAT exist: a) apart from the local signs (purulent secretions), there must be systemic signs and symptoms (fever and/or leukocytosis) as well as microbiological confirmation (positive tracheal cultures) for the diagnosis of VAT and, b) the ‘anatomical’ approach, requiring the presence of local signs (purulent secretions) and microbiologic ‘indication’ of infection (microorganisms in a Gram stain of tracheal secretions). The first approach basically differentiates VAT from VAP only by the absence (VAT) or presence (VAP) of radiological infiltrates [3,6,7,8,9,10,11,12,14,15,17]. Proponents of the ‘anatomical’ diagnostic approach believe no signs of systemic inflammation (e.g., fever, raised white bloods cells (WBC)) are needed [18,23]. Instead, diagnostic criteria for VAT include: presence of purulent secretions with a volume of at least 2mL per 4h with pathogens present on Gram stain [18,23]. The ‘anatomical’ approach is based on the concept that, as the proximal airway is anatomically and physiologically distinct from the lower airway, particularly in regard to vascular supply and surface area, it should be viewed as a unique compartment different than the alveolar space of the lung, susceptible to unique pathologies, such as VAT [3,18]. In both diagnostic approaches, the prerequisite and cornerstone of the criteria is the absence of new or progressive infiltrate in chest radiograph. Although given the limitations of mobile chest X-ray (CXR) for ICU patients in regard to sensitivity and specificity, it appears problematic to only distinguish VAP from VAT by recognition of new or progressive infiltrate on chest radiograph [24].

### 2.1. Under and Over-diagnosis of VAT

ICU patients are inherently complex, afflicted by multiple pathologies which confound the origin of systemic symptoms, which may lead to an under-diagnosis of VAT. In mechanically ventilated patients, both colonization and VAT will frequently result in positive microbiologic cultures of endotracheal aspirates. The new onset of systemic symptoms, in theory, could be considered an indication that the positive cultures relate to infection. However, the clinical picture is complicated, ICU patients with pre-existing systemic symptoms, such as those due to other sources of infection or a systemic inflammatory response syndrome (SIRS) response, may miss a VAT diagnosis if their systemic symptoms are attributed to other pathology or the positive cultures presumed to be colonization.

A 2018 multicenter cohort study analyzed ventilator-associated event (VAE) outcomes and their correlation to ventilator-associated pneumonia (VAP) and VAT diagnosis. This study utilized 2015 updated definitions: possible VAP (PVAP), infectious ventilator-associated complication (IVAC), and ventilator associated condition (VAC) and later compared them to the previous 2013 CDC classifications of VAP and VAT [16,25,26]. The study concluded that VAE classification only detected the most severe cases of sustained respiratory deterioration, while upwards of 25% of IVAC (VAT + VAP) are missed [16]. More importantly, 3 out of 4 VAT episodes did not even meet IVAC criteria [16]. The negative ramifications of VAT under-diagnosis could include an increased progression to VAP [6], accompanied by VAP’s associated increased morbidity, mortality, mechanical ventilation (MV), ICU length of stay (LOS), and healthcare costs [4].

Over-diagnosis and antibiotic treatment of VAT also presents its own issues, specifically in regard to increased adverse effects of treatment, along with selection pressure for microbiological resistance. In a post-hoc analysis from a prospective, observational trial on ICUs in the United States it was shown that 60% of patient diagnosed with SIRS were still treated with antibiotics [27]. This analysis, in agreement with the literature, concluded the lack of unambiguous clinical criteria to diagnose the septic patient as an explanation for the erroneous use of antibiotics [27]. Extrapolating this to the dilemma of colonization versus VAT and VAP diagnosis, it is necessary to find clear criteria for diagnosis and treatment to avoid this overuse of antibiotics we are seeing reflected in the murky waters of SIRS and sepsis delineation. There is significant support in the literature of colonization, VAT and VAP being on a continuum, therefore the issue lies with the accuracy of diagnosis of both conditions, which would allow the appropriate management to be delivered and mitigate over-diagnosis and inappropriate use of antibiotics.

### 2.2. Possible Diagnostic Adjuncts

The above diagnostic challenges highlight the need for adjunct and/or improved investigations, such as biomarkers or those which detect or exclude new or progressive lung infiltrates more accurately than CXR.

A prospective study evaluated the use of biomarkers in differentiating VAT from VAP. C-reactive protein (CRP) and procalcitonin (PCT) were used as biomarkers. The study found that the median CRP was elevated more significantly in VAP than VAT (18 mg/dL vs. 14 mg/dL, *p* < 0.001) and the median PCT was elevated more significantly in VAP than VAT (2.1 ng/dL vs. 0.64 ng/dL, *p* < 0.001) [28]. Unfortunately, there was significant overlap of both biomarker values making them poor metrics to differentiate VAT from VAP [28].

Fortuitously, studies investigating WBC gene upregulation (42 biomarkers) and four RNA biomarkers (SeptiCyte Lab, ImmuneExpress, Seattle, USA) in the acute phase response of the innate immune system have shown promise [29,30]. The innate immune response is non-specific and reacts similarly to SIRS (non-infectious) and sepsis (infectious) making it hard to demarcate the two. Sutherland et al. demonstrated that 42 molecular patterns could differentiate SIRS from healthy controls 92% of the time [30]. Additionally, they were able to accurately detect sepsis 86–92% (*p* < 0.002) of the time [30]. To ameliorate the studies limitations, McHugh et al. evaluated the SeptiCyte Lab genes across five different validation cohorts from ICU patients in Utrecht and Amsterdam [29]. These validation cohorts confirmed the original findings (AUC 0.95), concluded little bias towards SeptiCyte Lab score or ICU admission date, showed they retained accuracy in real-world settings (AUC = 0.85–0.93), across races (AUC = 0.92), gender (AUC = 0.9) and disease severity (as measured by SOFA and APACHE IV scores) [29]. In comparison to PCT (the only FDA cleared biomarker of sepsis), SeptiCyte Lab was repeatedly better at diagnosing sepsis (AUC = 0.84 vs. AUC = 0.89, respectively) [29]. The negative predictive value of a SeptiScore < 4 was calculated at 95% [29]. Taken all together, this indicates SeptiCyte Lab as a promising and time efficient (4–6 hours) test for differentiating SIRS from sepsis and therefore may also be useful in differentiating colonization from infection in VAT and VAP [29]. Further evaluation across more centers and in patients with more complex backgrounds (e.g., immunocompromised) is needed to further validate SeptiCyte Lab as a viable diagnostic adjunct.

Lung ultrasound (LUS) is becoming increasingly recognized as a reliable adjunct in the diagnosis of VAP [31,32,33,34]. In a systematic review LUS was shown to have a high sensitivity (94% CI, 92–96%) and specificity (96% CI, 94–97%) for the diagnosis of pneumonia [32]. Furthermore, it has been suggested that LUS may be particularly useful at identifying early pneumonia [34]. These results are especially encouraging given the other advantages LUS displays over CXR and thoracic CT, namely ease and speed of imaging and lack of ionizing radiation. If we accept the continuum theory and that LUS accurately detects early pneumonia better than CXR, we could then accept the absence of pneumonia on LUS as a better diagnostic metric than the absence of infiltrates on CXR—the current criteria from the CDC—to more accurately diagnose VAT. Further studies looking at both imaging modalities and comparing with thoracic CT would be needed to further elucidate the use of LUS in the diagnosis of VAT.

## 3. Treatment

Dilemmas on treatment approach are a direct and expected consequence of the dilemmas around the diagnosis of VAT. Current treatment obstacles include when to treat, antibiotics of choice, delivery method of antibiotics (intravenous, inhaled or both) and when to cease treatment(s).

### 3.1. Reasons to Treat VAT

One of the principal arguments in favor of the treatment of VAT is that no treatment or delayed antibiotic treatment would likely result in more severe clinical outcome otherwise avoided with earlier antibiotic treatment, including one or more of the following: progression to VAP, increased MV and ICU LOS, difficult weaning process from MV or increased costs [5,7,8,9,10,11,12,14,17,35].

The first randomised control study performed on the treatment efficacy of VAT demonstrated decreased ICU mortality for treated VAT patients versus patients not receiving antibiotics (18% vs. 47%; OR, 0.24, 95% CI 0.07–0.88), with the trial needing to be stopped early due to the apparent benefit of treatment [12]. In addition, VAT patients who had received antibiotics less frequently developed VAP (13% vs.47%; OR, 0.17, 95% CI, 0.04–0.70), although no difference in ICU LOS was found [12]. In this trial VAT was defined as fever (>38 °C) with no other recognizable cause, purulent sputum production, positive endotracheal aspirate culture yielding bacteria not present at intubation, and unremarkable radiology [12].

An observational study including patients with multidrug-resistant (MDR) pathogens showed no difference in mortality if VAT was appropriately treated, however did show an important decrease in progression to VAP (OR, 0.12, 95%; CI 0.02–0.59, *p* = 0.009) with a decrease in associated mortality [17]. Additionally, a multicenter prospective cohort of 2960 patients demonstrated that treating VAT was strongly associated with a favorable outcome, thought to be due to the protection it provides from developing VAP with its associated mortality (40% for VAP vs. 29% for VAT, *p* < 0.001) [9]. In that cohort, VAT was equally frequent to VAP (11% vs. 12%, respectively) and inappropriate antibiotic treatment was administered to 22% of the VAT patients [9]. Among patients with VAT, 12% further developed VAP, while appropriate antibiotic treatment was protective of progression to VAP (8% vs. 29% of those who received inappropriate treatment, *p* < 0.0001; crude OR, 0.21, 95% CI 0.11–0.41) [9]. Similar results have been seen in other studies relating to reduced progression to VAP with treatment of VAT [5,12,17] along with shorter duration of mechanical ventilation and shorter ICU stay [10].

MDR pathogens are increasingly implicated as causative agents in VAT within the last two decades, which may add further evidence of the importance of appropriate empirical antibiotic treatment for VAT, as is likewise acknowledged for VAP [7,8].

### 3.2. Reasons NOT to Treat VAT

Considering the almost universally high prevalence of VAT (up to 28.5% in neurosurgical ICU patients [8]), an ICU policy containing antibiotic prescription for all VAT episodes, with the primary aim of averting progression to VAP, would result in a significant increase in antibiotic consumption and likely exacerbate the antibiotic resistance epidemic (including difficult-to treat bacteria, such as *Pseudomonas aeruginosa* and *Acinetobacter baumannii*), whilst also increasing adverse effects associated with increased antibiotic use [36].

As a result, controversy remains regarding whether the treatment of all cases of VAT improves the patients’ outcomes, and whether there is a specific sub-group that should be targeted for treatment to maximize the treatment effect. A major hurdle to this approach is caused by difficulties and inconsistencies in defining VAT as were detailed previously, and the impact this has on clinical research into VAT [37].

The 2016 Infectious Diseases guidelines [19] on the management of adults with VAP recognized that IV antibiotic treatment of VAT may shorten the MV duration without affecting other outcomes such as mortality or ICU days, however did not recommend routine treatment due to inconsistent findings of the analyzed observational studies [3,7,10,11] and one randomized control trial analyzed to have a high risk of bias [12]. Accordingly, the Infectious Diseases Society of America (IDSA) guideline proposed that antibiotic treatment should not be considered in VAT patients despite possibly reducing MV duration, as the risks of increasing other adverse patient outcomes due to the increase in antibiotic prescription could be greater [19]. However, the IDSA emphasizes the need for individual patient evaluation for decision making, and that treatment should be initiated in severe cases [19]. It should be noted also, that at the time of this recommendation relevant data was sparse and in fact two of the three randomized control trials (RCTs) comparing antibiotic administration to placebo were deemed only indirectly related to the clinical question (due to differing definitions and evaluation of administration of inhaled antibiotics rather than IV) and ultimately excluded in the authors’ assessment [18,38].

### 3.3. Alternative Treatment Options

As an alternative to conventionally administered antibiotics, administration of inhaled aerosolized antibiotics has been proposed, either as treatment of VAT or to reduce the progression to VAP. The physiologic background that supports the administration of inhaled antibiotics in VAT is that the limited vascular supply of the airways compared to the abundant capillary bed of the lung alveoli leads to less surface exposed to thick purulent secretions and this might lead to the need of antibiotic concentration 15–20 times higher than minimum inhibitory concentration in order to be effective [23,39].

This type of treatment aims for less adverse effects compared to systematically delivered antibiotics, higher tissue antibiotic concentrations, supposed improved efficacy and less anticipated development of antibiotic resistance in the treated patients [38]. However, limited studies are available that focus specifically on VAT with regards to this method of treatment, with VAP being more widely studied [18]. In the first double blind, placebo-control RCT investigating the role of inhaled antibiotics for the treatment of VAT, aerosolized antibiotics adjuvant to intravenous treatment decreased the occurrence of VAP and facilitated weaning from MV, whilst also reducing antibiotic use and new antibiotic resistance occurrence [18]. In a second double-blind placebo-controlled study, aerosolized antibiotics eradicated MDR bacteria and reduced the occurrence of new antibiotic resistance [38].

A systematic review of six RCTs aimed to define the efficacy of inhaled antibiotics in the treatment of VAP and VAT [40]. In five out of the six included studies, intravenous antibiotics were concomitantly administered and the efficacy of treating with solely aerosolized antibiotics couldn’t be determined [18,40]. Divergent results were presented concerning delivery methods (e.g., ultrasonic or vibrating plate nebulizers), study protocols and outcome definitions [40]. The authors concluded that aerosolized antibiotics, either delivered solely or concomitantly with systemic antibiotics, are not sufficiently supported by available evidence [40].

In a review and meta-analysis performed on adults under mechanical ventilation, nebulized antibiotics significantly decreased the emergence of antibiotic resistance in VAT patients (RR, 0.18; 95% CI 0.05–0.64; I^2^ 0%), without decline in mortality or MV duration, with the overall rate of respiratory complications observed being 9% [41]. However, the authors emphasized the need for RCTs with less heterogeneous populations and more attention on standardized antibiotic delivery methods and safety issues [41].

In agreement with this, a guideline and position paper from the European Society of Clinical Microbiology and Infectious Diseases (ESCMID) on the use of nebulized antimicrobials for the treatment of respiratory infections in invasively mechanically ventilated adults recommends avoiding the use of nebulized antibiotics as adjuvants to intravenous antibiotics for the treatment of VAT due to low quality of evidence [42]. In addition, due to the absence of relevant trials, the authors recommend against the use of inhaled antibiotics as a sole therapy for VAT and a substitute of intravenous antibiotics [42]. A global online survey has been performed in 2017 to evaluate existing attitudes on aerosolized antibiotic prescription in mechanically ventilated patients in ICUs worldwide [43]. The reported data are in considerable deviation from the ESCMID 2017 guidelines since only 26.8% of the ICU physicians reported not using inhaled antibiotics and almost half of the participating physicians (49.4%) prescribed them for VAT [43]. The majority of the ICUs used the jet nebulizer and the most frequently prescribed antibiotics were colistin and amikacin [43].

In another review and meta-analysis of inhaled colistin monotherapy for respiratory tract infections (including VAT) [44], inhaled colistin administered as monotherapy without concomitant intravenous antibiotics achieved 33.8% pooled overall mortality (95% CI 24.6%–43.6%), 70.4% clinical success (58.5%–81.1%) and 71.3% eradication of Gram negative bacteria (57.6%–83.2%) in VAP or VAT [44]. Inhaled colistin was delivered as an exclusive antibiotic therapy in both studies, and clinical cure reached 80% and 95% in the two studies, while microbiological eradication was 40% and 95%, respectively. Neither VAT-attributed nor overall mortality was reported, and adverse events such as neurotoxicity and bronchospasm were recorded only by Maskin et al. and found to be 10% each [45]. Whilst promising, the authors stated that due to lack of controls in some of the selected studies, along with retrospective design and absence of standardized treatment parameters, the findings should be taken with caution and further well designed RCTs are necessary [44].

Further research on the role of aerosolized antibiotics is undoubtedly needed, with emphasis on specific indications, optimal dosage, delivery safety issues and aptitude to decrease systemic antibiotics’ duration, systemic adverse events and antibiotic resistance. Finding of the main VAT articles included in this review are summarized in the Table 1.

## 4. Conclusions and Future Perspectives

Given the vague nature of the most commonly applied diagnostic criteria for VAT, it is safe to assume that an ICU physician who decides to treat VAT empirically has little assistance from current clinical guidelines and available literature when determining whether a patient has VAT and not either a colonization or a true VAP episode. Whether VAT represents the intermediate step between lower airway colonization and VAP or a separate entity with distinct modes of acquisition and evolution, remains debatable. Future diagnosis and treatment hinder on the development of novel biomarkers and more accurate imaging techniques. SeptiCyte Lab or other (currently unknown) molecular signatures in conjunction with LUS may provide the diagnostic accuracy necessary to delineate colonization from VAT and VAP. Nonetheless, most studies have quite convincingly demonstrated that VAT is closely related to VAP in terms of frequency, microbial ecology and resistance patters, diagnostic procedures and associated morbidity and costs. On the contrary, in terms of mortality, a veritable association between VAT and ultimately unfavorable outcome has not been adequately shown. Nevertheless, if one accepts the relevance between untreated VAT and progression to VAP, it is reasonable to assume that overall avoidance of treating VAT will incur more frequent cases of VAP, possibly leading to increased mortality.

Until a consensus definition of VAT is reached and guidelines on VAT management are either more widely implemented or ultimately overruled, partially or completely, an individualized case by case diagnostic and treatment approach of VAT should be the paradigm. To accomplish this tailored approach of every VAT episode, apart from the patient’s individual characteristics, medical conditions, and comorbidities, ICU physicians need to be aware of certain factors, including local epidemiological data on the prevailing pathogens and MDR rates, pertinence of the in-use diagnostic procedures, the overall infection prevention and control policy on VAT and VAP, and the overall antibiotic policy. Lastly, as other ICU-acquired infections, the VAT treatment approach should be regularly evaluated for its overall impact on the entire antibiotic use and antibiotic resistance rates in ICUs, especially those with high MDR prevalence.

Future research into the topic must include sufficiently powered studies aiming to evaluate the actual effect of VAT treatment on clinical outcomes. Such studies should incorporate stringent and widely accepted definition criteria, along with accurate and timely diagnostic tests and updated policies on the most appropriate antibiotic regimens (agents, doses, routes, duration).

## Figures and Tables

**Table 1:** Summary of the main finding of studies on VAT included in this review.

Article	Type	Cohort	Transition of VAT to VAP	Impact on MV duration, ICU-LOS, Mortality	Pathogens
[3]	Prospective, observational, single centre cohort study	1889 patients	VAT: 201/1889 (10.6%)	VAT significantly increased MV duration & ICU-LOS in both medical and surgical patients, BUT non-significant difference in mortality Mortality only significantly improved with VAT treatment in medical patients. All other outcomes: not significant	VAT - *Pseudomonas* = 72 - *Acinobacter* = 61 - *Klebsiella* = 10 - *Serratia* = 13 - *E. coli* = 9 - MRSA = 38 - MSSA = 8
[15]	Prospective, single centre,	356 patients, all undergoing major cardiac procedures	Frequency of VAP = 7.87% (28/356) Frequency of VAT = 8.15% (29/356) -5/29 progressed to VAP	ICU-LOS -VAP: significant increase in mean length of stay (*p* < 0.05) -VAT: Insignificant difference MV duration -Significant longer in combined VAT/VAP group compared to no infection (*p* < 0.0001) Mortality -Significantly higher in VAP (16/28 = 57.1%) and VAT (6/29 = 20.7%) than non-colonized patients	VAP (28 total) - *Pseudomonas* =5 - *S. aureus* = 5 - *Serratia* = 3 - Polymicrobial = 3 VAT (29 total) - *H. influenzae* = 9 - *Moraxella catarrhalis* = 2 - Polymicrobial = 2
[10]	Prospective, observational, case-control study	1131 patients	VAT: 103/1131 (9.1%) 11/103 (10.6%) progressed to VAP 81/103 were control matched	ICU-LOS -Significantly longer in VAT (*p* = 0.022) MV duration -Significantly longer in VAT (*p* = 0.015) ICU mortality: -No significant difference	VAT (n = 81) - *Pseudomonas* = 32 - *Serratia* = 6 - *H. influenzae* = 5 - *Enterobacter* = 7 - MRSA = 19 - MSSA = 6
[11]	Retrospective, single centre, case-control study	792 patients	VAT: 70/792 (8%) 7/70 progressing to VAP	MV duration (*p* = 0.001) & ICU-LOS (*p* = 0.001) significantly longer in VAT ICU mortality showed no significant difference	VAT - *Pseudomonas* = 30 - *Acinetobacter* = 16 - *Serratia* = 6 - MRSA = 10 - MSSA = 5
[18]	Phase III, double-blinded placebo-controlled, single centre study	43 patients	n/a	Number of MV-free days not significantly different. Mortality between the two groups were not significantly different.	n/a
[12]	Prospective, multicentre, randomized controlled, unblinded study	58 patients randomly assigned; 44 included in the analysis	Progression to VAP 20/58 progressed to VAP	Progression to VAP, MV duration & mortality significantly improved with antibiotics treatment of VAT, causing the study to be terminated early	VAT - *Pseudomonas* = 32% - MSSA = 3 - MRSA = 3 - *E. coli* = 3 - *Proteus mirabilis* = 3
[7]	Single centre, prospective, observational study	2060 patients admitted to ICU over 1 year; 111 were identified as having VAP or VAT	VAP: 83/111 (74.8%) VAT: 28/111 (25.2%) Progressed to VAP in 9 patients (32.1%)	No significant difference between ICU-LOS or MV duration between both VAT and VAP groups. Mortality was not an outcome measured	VAP - MRSA = 10 - MSSA = 9 - *S. pneumoniae* = 5 - *Acinetobacter* = 10 - *Pseudomonas* = 11 - *Enterobacter* = 6 VAT - MRSA = 6 - MSSA = 4 - *Acinetobacter* = 5 - *Pseudomonas* = 3 - *H. influenzae* = 3
[6]	Prospective, single ICU study	188 patients	VAP & VAT: 43/188 (23%) 6 with VAT progressed to VAP (29%)	ICU-LOS -VAT + VAP: both significantly longer (*p* < 0.02 & *p* = 0.02) MV duration -VAT: Significantly longer (*p* = 0.01) -VAP: Significantly longer (*p* = 0.01) Mortality -VAT + VAP = no difference	VAP (28) - MRSA = 6 - MSSA = 11 - *E. coli* = 3 VAT (21) - MRSA = 5 - MSSA = 8 - *P. aeruginosa* = 4
[8]	Prospective, observational, single centre cohort study	236 patients	VAP: 78/236 (33.1%) VAT: 42/236 (18%) 7/42 later progressed to VAP with same organism	ICU-LOS -VAT: prolonged ICU stay (*p* = 0.007) MV duration -VAT: prolonged compared to no infection (*p* = 0.002), but significantly less than VAP (*p* = 0.004) Mortality -VAT: no increased mortality	VAP - *Acinobacter* = 40% VAT - *Acinobacter* = 20% - *Pseudomonas* = 10% - *Klebsiella* = 8%
[14]	Prospective, single centre, observational study	287 patients ventilated for >48hrs in ICU	Suspected (s) VARI= 77/287 -sVAT = 48 (62%) -sVAP = 29 (38%)	ICU-LOS -sVAP = significantly increased (*p* < 0.01) -sVAT = significantly increased (*p* < 0.003)	n/a
[17]	Prospective observational multicentre study	1501 patients	VAT: 122/1501 (7.1%) 17 (13.9%) progressed to VAP	n/a	VAT - *Pseudomonas* = 44 - *Enterobacter* = 12 - *E. coli* = 6 - *Acinetobacter* = 14 - *Klebsiella* = 13 - MRSA 12 - MSSA 13
[9]	Multicentre, prospective observational study	2960 patients	VAP: 269/2960 (12%) VAT: 320/2960 (11%) 39 progressed to VAP	ICU-LOS -significantly increased in VAT + VAP compared to no respiratory infection MV duration -significantly increased in VAT + VAP compared to no respiratory infection	VAP - *S. pneumoniae* = 24 - *Stenotrophomonas* = 12 - MSSA = 80 - MRSA = 8 - *Pseudomonas* = 89 - *Klebsiella* = 53 - *E. coli* = 40 - *Enterobacter* = 46 VAT - *S. pneumoniae* = 16 - *Stenotrophomonas* = 19 - MRSA= 8 - MSSA 66 - *Pseudomonas* = 79 - *Klebsiella* = 48 - *H. nfluenzae* = 32 - *E. coli* = 37 - *Enterobacter* = 35

Abbreviations: MSSA (methicillin-sensitive S.aureus); MRSA (methicillin-resistant S.aureus); MV (mechanical ventilation); LOS (length of stay); VARI: ventilator-associated lower respiratory infection; VAT: ventilator-associated tracheobronchitis; VAP: ventilator-associated pneumonia; ICU-LOS: intensive care unit length of stay; MV: mechanical ventilation.
